# A 3-Tier AI Model for COVID-19 Triage Using Pharyngeal Images: Algorithm Development and Validation

**DOI:** 10.2196/87705

**Published:** 2026-07-20

**Authors:** Sho Okiyama, Tomonori Aoki, Memori Fukuda, Yuji Ariyasu, Saho Kameyama

**Affiliations:** 1 Department of Digital Health, Institute of Medicine, University of Tsukuba, 1-1-1 Tennodai, Tsukuba, Ibaraki, 305-8571, Japan, +81 90 6522 4161; 2 Aillis, Inc, Tokyo, Japan; 3 Flinders Health and Medical Research Institute, College of Medicine and Public Health, Flinders University, Adelaide, Bedford Park, SA, Australia

**Keywords:** COVID-19, artificial intelligence, AI, machine vision, decision support, pharyngeal images

## Abstract

**Background:**

SARS-CoV-2 remains a common cause of acute respiratory illness; however, symptom-based triage poorly discriminates it from other febrile conditions. A recently developed artificial intelligence (AI)–powered pharyngeal camera acquires pharyngeal images and clinical data to assist in influenza diagnosis; leveraging this workflow, we evaluated an adjunct AI algorithm (COVID-19-AI) that reports high, medium, or low suspicion to guide whether SARS-CoV-2 testing should subsequently be performed.

**Objective:**

This study aimed to report diagnostic accuracy outcomes and clinical utility of the COVID-19-AI as a triage support tool.

**Methods:**

We conducted a performance evaluation using a prospectively collected multicenter dataset from 26 Japanese institutions between December 2023 and March 2024. Patients with suspected influenza or COVID-19 were eligible. The COVID-19-AI algorithm, a stacked ensemble of a Swin Transformer and boosting models, was developed using pharyngeal images combined with routine clinical variables from 2133 patients, and it produced a 3-tier output. Classification thresholds were predefined to optimize clinical rule-out and rule-in utilities. Diagnostic performance was assessed in 696 independent patients against centralized reverse transcription polymerase chain reaction–confirmed SARS-CoV-2 infection under 2 prespecified operating criteria: inclusive (high or medium=positive and low=negative) and strict (high=positive and medium or low=negative). A subanalysis stratified accuracy by time from symptom onset (12-hour bins to 72 hours).

**Results:**

Among 696 analyzed participants (all Asian), 247 (35.5%) had reverse transcription polymerase chain reaction–confirmed SARS-CoV-2 infection. The COVID-19-AI categorized 12.4% (n=86), 72.8% (n=507), and 14.8% (n=103) patients as high, medium, and low suspicion, respectively. Under the inclusive criteria, sensitivity of COVID-19-AI was 93.9% (95% CI 90.4%‐96.4%), specificity was 19.6% (95% CI 16.1%‐23.5%), and negative predictive value was 85.4% (95% CI 77.6%‐91.3%). Under the strict criteria, sensitivity was 24.7% (95% CI 19.6%‐30.4%), specificity was 94.4% (95% CI 92.0%‐96.3%), and positive predictive value was 70.9% (95% CI 60.7%‐79.8%). Across 12-hour onset strata, sensitivity under the inclusive criteria remained ≥92.0% and specificity under the strict criteria remained ≥83.3%; no pronounced temporal trend was observed. Additionally, an integrated model using both pharyngeal images and clinical variables (area under the receiver operating characteristic curve [AUROC] 0.78) outperformed models using only clinical variables (AUROC 0.75) or images alone (AUROC 0.71); feature importance analysis further confirmed that pharyngeal image information was the most influential individual predictor, providing greater predictive value than any single clinical variable.

**Conclusions:**

Embedded within AI-powered pharyngeal camera workflows, a 3-tier AI suspicion output enables complementary operating behaviors—high sensitivity to rule out COVID-19 (inclusive criteria) and high specificity to support immediate infection control measures (strict criteria), although these criteria involve inherent trade-offs with low specificity and low sensitivity, respectively. Performance stability across onset times suggests robustness to symptom chronology, offering a standardized tool for clinical triage.

## Introduction

SARS-CoV-2 continues to circulate globally and remains a persistent cause of acute respiratory illness among patients presenting with respiratory symptoms [1,2]. Differentiating SARS-CoV-2 from other common viral infections, including influenza and adenovirus, remains challenging because of substantial symptom overlap and its diverse systemic and neurological manifestations [3-5]. Accordingly, when decisions regarding SARS-CoV-2 testing are based solely on symptoms, triage may fail to identify some individuals with COVID-19 for testing while unnecessarily referring low-risk individuals for testing, resulting in increased patient burden and inefficient use of health care resources [6-12]. Although adding information from blood tests or computed tomography can improve predictive accuracy [13,14], lower-burden sources of additional signal are preferable. While traditional nasopharyngeal swabbing remains the standard clinical procedure for COVID-19 testing, its invasive nature and procedural discomfort, combined with the time required for results, can create bottlenecks in high-volume triage settings.

Recent case series and cross-sectional studies have suggested that pharyngeal findings may be observed in patients with COVID-19 [15-19]. Although this examination is simple, its interpretation is subjective and varies with the clinician’s experience. Standardization is also required to mitigate patient-specific factors such as macroglossia, limited mouth opening, or a strong gag reflex, which can hinder consistent visual assessment. Minimizing this variability and standardizing assessment are therefore warranted.

To address these issues, we sought to leverage a recently approved artificial intelligence (AI)–powered medical device which acquires pharyngeal images and integrates clinical information for influenza diagnosis [20]. Building on the existing workflow, we combined pharyngeal images with routine clinical data to estimate the likelihood of SARS-CoV-2 infection and developed an adjunct AI model (“COVID-19-AI”) that outputs high, medium, or low suspicion. This system is designed as a tool to facilitate clinical decision-making regarding whether further invasive SARS-CoV-2 testing should be prioritized or may be deferred (Multimedia Appendix 1). In this study, we evaluate the performance of this 3-tier risk stratification and its clinical utility in optimizing triage workflows.

## Methods

### Study Design and Participants

We conducted a noninterventional performance evaluation using a multicenter, prospectively collected observational dataset from 26 medical institutions in Japan (December 2023 to March 2024; registered in the UMIN Clinical Trials Registry: UMIN000052896). These institutions were distributed nationwide across 5 geographic regions (from Hokkaido to Kyushu), spanning internal medicine (n=11, 42%), otolaryngology (n=8, 31%), and pediatrics (n=7, 27%). Detailed characteristics are summarized in Multimedia Appendix 2.

The inclusion criteria were as follows: patients with suspected influenza or COVID-19 who underwent testing for both conditions; provided written consent (self or surrogate); met ≥1 of the following clinical triggers—fever ≥37.0 °C, systemic symptoms (eg, joint pain, muscle pain, headache, tiredness, and appetite loss), respiratory symptoms (eg, cough, sore throat, and nasal congestion), or clinician-judged suspicion of influenza or COVID-19 (eg, close contact); and were aged ≥6 years at consent. The exclusion criteria included impaired consciousness or breathing disorders; moderate or severe gingival or dental disease or loose teeth that may be aggravated by the research equipment; trauma, damage, or surgery to the maxillofacial, dental, oral, or pharyngeal regions that might be aggravated by the device; recurrent vomiting; or investigator-determined ineligibility.

Pharyngeal images and clinical information from 2133 patients were used to train a COVID-19-AI model (Multimedia Appendix 1). To evaluate the model, 700 independent patients were enrolled. Of these, 696 (99.4%) were included in the full analysis set after 4 records were excluded due to out-of-window entry dates.

### Collected Variables

**Figure 1.**
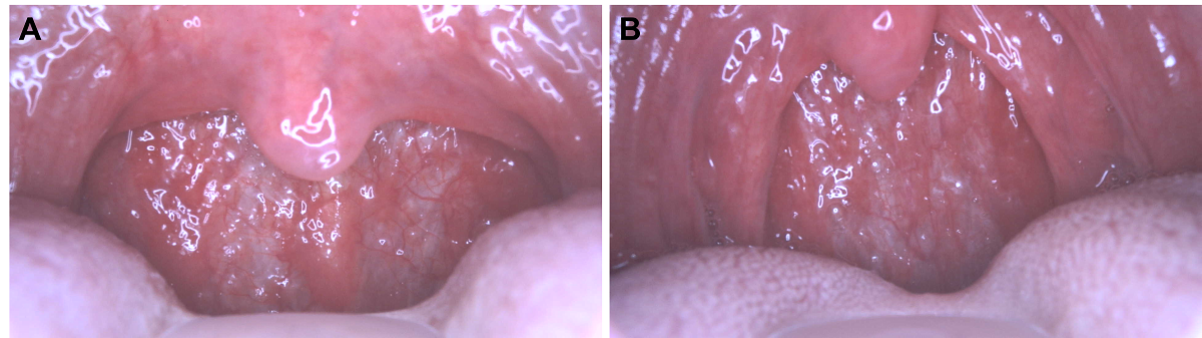
Representative pharyngeal images of patients with COVID-19. The images were obtained from a multicenter, prospective performance evaluation of the COVID-19-AI triage tool. The study involved 696 Asian patients presenting with fever or respiratory symptoms at 26 Japanese institutions between December 2023 and March 2024. (A) A 64-year-old man (time from symptom onset: 42.3 hours) and (B) a 52-year-old man (time from symptom onset: 15.0 hours).

In addition to pharyngeal images (Figure 1), the following clinical variables were obtained: age, sex, race, vaccination history, close contact history, time from the first symptom onset, use of antipyretics, vital signs at the visit (body temperature, pulse rate, and oxygen saturation), presenting symptoms (joint pain, muscle pain, headache, tiredness, appetite loss, chill, sweating, cough, sore throat, nasal discharge, nasal congestion, respiratory inflammation, abdominal pain, vomiting or diarrhea, sputum production, dyspnea, smell disorder, and taste disorder), and pharyngeal examination features (tonsillar white moss, redness, swelling or exudates, and tender anterior cervical lymphadenopathy). Furthermore, reverse transcription polymerase chain reaction (RT-PCR) and rapid immunochromatographic antigen detection test results for both influenza and COVID-19 were obtained. To ensure a standardized reference across all 26 sites, centralized RT-PCR was performed using residual liquid from rapid antigen test kits, which was frozen and transported to a central laboratory (Mediford Corporation, Tokyo, Japan) for analysis. The RT-PCR result for COVID-19 served as the reference (gold) standard for the diagnosis of SARS-CoV-2 infection. While the initial AI model was trained using high-quality positive labels defined by dual positivity (both RT-PCR and rapid antigen test positive)—with discordant results categorized as negative to minimize noise from cases with low viral loads—the final performance evaluation strictly followed the RT-PCR–confirmed status as the sole reference in accordance with current clinical needs and consultations with the Pharmaceuticals and Medical Devices Agency. In this evaluation, all RT-PCR–positive cases, including those with discordant antigen test results, were categorized as true positives to rigorously validate the model’s utility in real-world clinical settings. All clinicians were blinded to the AI model’s output during the prospective data collection phase to ensure that physical examinations and RT-PCR testing decisions remained independent of the model’s predictions.

### Outcomes and Analysis

The outcome measure was the diagnostic accuracy of COVID-19-AI against the RT-PCR reference, expressed as sensitivity and specificity. The COVID-19-AI produces a 3-tier output (high, medium, or low) from pharyngeal images and clinical variables (Multimedia Appendix 1). Performance was evaluated under 2 prespecified operating criteria: inclusive (high or medium=positive and low=negative) and strict (high=positive and medium or low=negative). In a subanalysis, accuracy was further examined across time from symptom onset using 12-hour strata up to 72 hours, with RT-PCR–referenced sensitivity and specificity estimated under both operating criteria.

### Ethical Considerations

This study was conducted according to the guidelines of the Declaration of Helsinki and was approved by the central institutional review board of Nihonbashi Sakura Clinic (approved on November 22, 2023, for prospective data collection and on August 21, 2024, for performance evaluation). Written informed consent for research participation and reporting was obtained from all participants or their legally authorized representatives. Patient data were anonymized at the study-ID level prior to transfer to the analysis team. Each participant received a ¥1500 (¥1=US$0.006 as of June 15, 2026) gift card as compensation for their participation. Furthermore, we ensured that no individual participant is identifiable from the images or any other information presented in this manuscript.

## Results

### Patient Characteristics

Among 696 patients included in the validation of the COVID-19-AI model, 48.7% (n=339) were male, and the mean age was 32.2 years (SD 19.1 years; Table 1). The mean time from symptom onset to presentation was 35.9 hours (SD 30.7 hours). The mean visit temperature was 37.45 °C (SD 0.96 °C), and the mean oxygen saturation was 97.9% (SD 1.3%). All participants were of Asian race. By RT-PCR, 247 (35.5%) patients tested positive for COVID-19 and 449 (64.5%) tested negative.

**Table 1.** Patient characteristics of the full analysis set (N=696): prospectively collected data of Asian patients presenting with fever or respiratory symptoms at 26 Japanese institutions between December 2023 and March 2024.

Characteristics	Value
Age (years), mean (SD)	32.2 (19.1)
Sex, n (%)	
Male	339 (48.7)
Female	357 (51.3)
Race, n (%)	
Asian	696 (100.0)
Others	0 (0.0)
Time from onset (hours), mean (SD)^a^	35.9 (30.7)
Highest BT^b^ before visit (°C), mean (SD)	38.28 (0.93)
Close contact with febrile patients within 14 days, n (%)	182 (26.1)
Time from close contact (hours), mean (SD)^c^	98.0 (77.2)
Close contact with influenza patients within 3 days, n (%)	74 (10.6)
Time from close contact (hours), mean (SD)^c^	53.3 (36.9)
Close contact with COVID-19 patients within 14 days, n (%)	88 (12.6)
Time from close contact (hours), mean (SD)^c^	83.9 (60.4)
Recent influenza vaccination, n (%)	234 (33.6)
Recent COVID-19 vaccination, n (%)	497 (71.4)
Use of antipyretics, n (%)	335 (48.1)
Time from use of antipyretics (hours), mean (SD)^d^	12.7 (9.8)
Subjective symptoms, n (%)	
Joint pain	200 (28.7)
Muscle pain	94 (13.5)
Headache	396 (56.9)
Tiredness	433 (62.2)
Appetite loss	116 (16.7)
Chill	321 (46.1)
Sweating	87 (12.5)
Cough	403 (57.9)
Sore throat	462 (66.4)
Nasal discharge	374 (53.7)
Nasal congestion	200 (28.7)
Abdominal pain	49 (7.0)
Vomiting	32 (4.6)
Diarrhea	48 (6.9)
Sputum	211 (30.3)
Dyspnea	43 (6.2)
Smell disorder	7 (1.0)
Taste disorder	10 (1.4)
Objective findings	
BT at visit (°C), mean (SD)	37.45 (0.96)
Pulse rate (beats/min), mean (SD)	97.8 (18.5)
Oxygen saturation (%), mean (SD)	97.9 (1.3)
Tonsillitis, n (%)	51 (7.3)
Tonsillar white moss, n (%)	11 (1.6)
Tonsillar redness, n (%)	240 (34.5)
Tonsillar swelling or exudate, n (%)	14 (2.0)
Tender anterior cervical lymphadenopathy, n (%)	7 (1.0)
Influenza test (rapid antigen test), n (%)	
Positive	201 (28.9)
Negative	495 (71.1)
Influenza test (RT-PCR)^e^, n (%)	
Positive	233 (33.5)
Negative	463 (66.5)
COVID-19 test (rapid antigen test), n (%)	
Positive	202 (29.0)
Negative	494 (71.0)
COVID-19 test (RT-PCR), n (%)	
Positive	247 (35.5)
Negative	449 (64.5)

a Time from the first symptom onset to the study site visit.

b BT: body temperature.

c Time from close contact to the study site visit.

d Time from the last use of antipyretics to the study site visit.

e RT-PCR: reverse transcription polymerase chain reaction.

### Main Analyses

Among the total 696 patients, the COVID-19-AI categorized 12.4% (n=86) as “high” suspicion, 72.8% (n=507) as “medium” suspicion, and 14.8% (n=103) as “low” suspicion.

Under the inclusive operating criteria (high or medium vs low), sensitivity was 93.9% (95% CI 90.4%‐96.4%) and specificity was 19.6% (95% CI 16.1%‐23.5%). Thus, the inclusive criteria captured nearly all RT-PCR–positive COVID-19 cases, consistent with a sensitivity-oriented triage role yielding a high negative predictive value (85.4%, 95% CI 77.6%‐91.3%), which is highly effective for ruling out COVID-19 when a low suspicion output is returned (Table 2).

**Table 2.** Sensitivity and specificity of the artificial intelligence model compared with the reference standard diagnosis of SARS-CoV-2 infection (N=696): prospectively collected data of Asian patients presenting with fever or respiratory symptoms at 26 Japanese institutions between December 2023 and March 2024.

	SARS-CoV-2 infection based on RT-PCR^a^ (reference standard)		
Inclusive criteria^b^^c^			
Sensitivity, % (95% CI)	93.9 (90.4‐96.4)		
Specificity, % (95% CI)	19.6 (16.1‐23.5)		
Strict criteria^d^^e^			
Sensitivity, % (95% CI)	24.7 (19.6‐30.4)		
Specificity, % (95% CI)	94.4 (92.0‐96.3)		

a RT-PCR: reverse transcription polymerase chain reaction.

b AI-positive under the inclusive criteria (high or medium; total n=593): true positive, n=232; false positive, n=361; positive predictive value=39.1% (95% CI 35.3%-43.1%).

c AI-negative under the inclusive criteria (low; total n=103): false negative, n=15; true negative, n=88; negative predictive value=85.4% (95% CI 77.6%-91.3%).

d AI-positive under the strict criteria (high; total n=86): true positive, n=61; false positive, n=25; positive predictive value=70.9% (95% CI 60.7%-79.8%).

e AI-negative under the strict criteria (medium or low; total n=610): false negative, n=186; true negative, n=424; negative predictive value=69.5% (95% CI 65.8%-73.1%).

Under the strict criteria (high vs medium or low), sensitivity was 24.7% (95% CI 19.6%‐30.4%) and specificity was 94.4% (95% CI 92.0%‐96.3%). As anticipated, the strict criteria markedly increased specificity, resulting in a positive predictive value of 70.9% (95% CI 60.7%‐79.8%). This makes the high-suspicion output useful for prioritizing infection control measures (Table 2).

### Subanalysis: Time-Since-Onset Stratification

With the inclusive criteria, sensitivity remained high (≥92.0% across all 12-hour bins), while specificity was relatively low (approximately 20%). Under the strict criteria, specificity remained high (≥83.3% across all bins) at the expense of lower sensitivity (approximately 25%; Table 3 and Table 4). Neither set of criteria showed a pronounced trend with onset time.

**Table 3.** Sensitivity of the rapid antigen test and the artificial intelligence (AI) model compared with the reference standard diagnosis of SARS-CoV-2 infection, stratified by time from symptom onset (12-hour bins)^a^.

Time from symptom onset (hours)	RT-PCR^b^ (reference standard)	Rapid antigen test		AI model (inclusive criteria)		AI model (strict criteria)	
	Positive, n	Positive, n	Sensitivity, % (95% CI)	Positive (high or medium), n	Sensitivity, % (95% CI)	Positive (high), n	Sensitivity, % (95% CI)
0‐12	20	14	70.0 (47.7-86.8)	19	95.0 (77.7-99.7)	4	20.0 (6.7-41.5)
12‐24	75	60	80.0 (69.8-87.9)	69	92.0 (84.1-96.7)	17	22.7 (14.3-33.1)
24‐36	63	56	88.9 (79.3-95.0)	61	96.8 (89.9-99.5)	21	33.3 (22.5-45.6)
36‐48	54	42	77.8 (65.3-87.4)	52	96.3 (88.3-99.4)	14	25.9 (15.6-38.8)
48‐60	12	9	75.0 (45.9-93.2)	12	100.0 (77.9-100.0)	2	16.7 (2.9-45.1)
60‐72	6	6	100.0 (60.7-100.0)	6	100.0 (60.7-100.0)	2	33.3 (6.0-73.8)

a Direct head-to-head comparisons of sensitivity or specificity between the rapid antigen test and the artificial intelligence criteria are inappropriate, as these tools achieve their performance at vastly different trade-off levels.

b RT-PCR: reverse transcription polymerase chain reaction.

**Table 4.** Specificity of the rapid antigen test and the artificial intelligence (AI) model compared with the reference standard diagnosis of SARS-CoV-2 infection, stratified by time from symptom onset (12-hour bins)^a^.

Time from symptom onset (hours)	RT-PCR^b^ (reference standard)	Rapid antigen test		AI model (inclusive criteria)		AI model (strict criteria)	
	Negative, n	Negative, n	Specificity, % (95% CI)	Negative (low), n	Specificity, % (95% CI)	Negative (medium or low), n	Specificity, % (95% CI)
0‐12	50	49	98.0 (90.5‐99.9)	9	18.0 (9.2‐30.5)	48	96.0 (87.4‐99.3)
12‐24	142	141	99.3 (96.6‐100.0)	22	15.5 (10.2‐22.2)	136	95.8 (91.4‐98.3)
24‐36	95	95	100.0 (96.9‐100.0)	25	26.3 (18.2‐35.8)	92	96.8 (91.6‐99.2)
36‐48	64	63	98.4 (92.5‐99.9)	8	12.5 (6.0‐22.4)	56	87.5 (77.6‐94.0)
48‐60	37	37	100.0 (92.2‐100.0)	10	27.0 (14.6‐42.9)	35	94.6 (83.3‐99.1)
60‐72	12	12	100.0 (77.9‐100.0)	3	25.0 (6.9‐54.1)	10	83.3 (54.9‐97.1)

a Direct head-to-head comparisons of sensitivity or specificity between the rapid antigen test and the artificial intelligence criteria are inappropriate, as these tools achieve their performance at vastly different trade-off levels.

b RT-PCR: reverse transcription polymerase chain reaction.

## Discussion

### Principal Findings

This multicenter evaluation demonstrates that a 3-tier COVID-19-AI model embedded in the AI-powered pharyngeal camera workflow can deliver complementary operating behaviors: very high sensitivity (93.9%) under the inclusive criteria, with the intended use of ruling out COVID-19 as a triage tool, and very high specificity (94.4%) under the strict criteria, with the intended use of guiding immediate infection control measures (Table 2). Stability across 12-hour onset strata suggests robustness to symptom chronology, a known determinant of index test performance in infectious disease triage [21,22]. Feature importance analysis indicates that incorporating pharyngeal images materially improves the prediction of SARS-CoV-2 infection, with pharyngeal image information providing greater predictive value than any single clinical variable (Figure S1 in Multimedia Appendix 1). Furthermore, an ablation study confirmed that the integrated model using both pharyngeal images and clinical information (area under the receiver operating characteristic curve [AUROC] 0.78) significantly outperformed models using only clinical information (AUROC 0.75; *P*<.001) or only pharyngeal images (AUROC 0.71; *P*<.001; Figure S2 in Multimedia Appendix 1). The diagnostic value of pharyngeal imaging is corroborated by recent evidence demonstrating the high accuracy of deep learning models in diagnosing respiratory infections and evaluating upper airway conditions [23-26].

### Clinical Utility of AI-Driven Triage

Estimating pretest probability from a stratified 3-tier output allows for clear, action-oriented clinical decision-making. By providing instantaneous, noninvasive risk stratification upon a patient’s arrival, this tool enables the immediate categorization of patients, thereby streamlining the diagnostic pathway. Cases with the “low” output, supported by the high sensitivity of the inclusive criteria (93.9%), could be appropriately considered as COVID-19 excluded without additional testing (eg, rapid antigen testing or RT-PCR). Furthermore, our simulation across different COVID-19 prevalence settings demonstrates that the inclusive criteria maintain an exceptionally high negative predictive value (96.7%‐98.4% at 5%‐10% prevalence; Multimedia Appendix 3). This indicates that in general screening populations where COVID-19 is less common, a “low” suspicion result provides a highly reliable rule-out signal. Such a high degree of safety allows clinicians to confidently prioritize other differential diagnoses for low-risk patients, optimizing patient comfort. Conversely, cases with a “high” suspicion output, supported by the high specificity of the strict criteria (94.4%), enable immediate routing for infection control. Although the positive predictive value (70.9%) may not be sufficient for a definitive diagnosis, this high-suspicion signal provides a pragmatic basis for presumptive isolation, substantially enhancing facility safety compared to universal testing.

### Resource Stewardship and Implementation

From a practice and resource stewardship standpoint, because medical and human resources are finite, it is not always feasible to perform RT-PCR testing in all potentially eligible patients. In our study, the inclusive criteria resulted in a 14.8% reduction in the testing burden compared to universal testing. While the “high” and “low” categories offer decisive signals, 72.8% of cases are labeled as “medium” suspicion, reflecting the reality of intermediate-risk populations in clinical triage. These medium cases require clinician judgment for additional testing based on clinical priorities and local testing capacity. Specifically, testing should be prioritized for the medium cases where tolerance for false negatives (ie, missed cases) is low. Conversely, testing may be deferred or excluded when patient burden, cost, or local testing capacity are major constraints. Thus, the 3-tier score serves as a decisive reference criterion to support rapid clinician judgment in dynamic workflows. Notably, this adjunct AI model has recently received regulatory approval (October 2025) from Japan’s Ministry of Health, Labour and Welfare (30400BZX00101000) for integration into the NODOCA device (Aillis, Inc). In this approved implementation, the high, medium, and low outputs are officially positioned as “detected,” “detection suspended,” and “undetected,” respectively.

### Advantages Over Nasopharyngeal Swabbing

Regarding patient experience and procedural quality, clinical data using this pharyngeal camera confirmed that image capture at the pharynx resulted in significantly less procedural pain than nasopharyngeal swabbing (median numerical rating scale 1 vs 3), supporting its use as a lower-burden diagnostic pathway [27]. Consequently, triaging out low-risk presentations can reasonably reduce patients’ exposure to the discomfort—and occasional complications—associated with nasopharyngeal swab testing. Furthermore, this noninvasive and instantaneous approach eliminates the procedural exposure risks and the mandatory turnaround time inherent to rapid antigen testing. Notably, previous research using the same pharyngeal camera for influenza diagnosis demonstrated that the AI system was effective in identifying positive cases at an earlier stage of illness than rapid antigen testing [28]. This might reflect a fundamental difference in diagnostic methodologies: conventional antigen tests detect pathogen viral load, whereas this pharyngeal AI evaluates the host immune response. Consequently, this AI-powered pharyngeal triage may serve as a valuable complement during the early phase of infection when antigen sensitivity is limited, although larger-scale validation for COVID-19-AI is required given our study’s limited early-onset sample size. Moreover, sampling quality and subsequent positivity rates have been shown to vary with collector experience [29], suggesting that narrowing test eligibility allows limited expert staff to focus on higher-yield cases, which may in turn help reduce the risk of false negatives.

### Limitations

Our study has some limitations. First, this study was conducted exclusively in Japan across 26 institutions, and the evaluation population was entirely Asian. As pharyngeal anatomy and mucosal inflammatory responses may vary across ethnic groups and clinical practices differ by country, the generalizability of our results to non-Asian populations requires caution and independent validation. Second, we did not collect several potentially informative predictors for confirming true SARS-CoV-2 infection, which may have limited the model’s performance. For example, our AI-assisted pharyngeal camera is not designed to capture tongue images, even though emerging evidence indicates that tongue imaging features can contribute to predictive performance [30]. Third, our data split was performed at the patient level, meaning the 26 institutions were shared between the training and test sets. While this allowed the model to incorporate diverse clinical environments (eg, lighting and backgrounds), future studies in entirely independent institutions are needed to further confirm its generalizability. Fourth, the use of 10 different rapid antigen test kits and varying sampling techniques across 26 institutions may have introduced intersite heterogeneity; however, such diversity reflects real-world clinical settings. Although centralized RT-PCR was used to provide a standardized reference, the use of frozen residual liquid from the rapid antigen test kits may have impacted analytical sensitivity and potentially led to the misclassification of cases with very low viral loads. Fifth, the small sample size in the late-onset window (eg, n=6 for 60‐72 hours) results in wide CIs, and our findings regarding performance stability should be interpreted with caution. Sixth, patients aged <6 years were excluded due to the difficulty of maintaining stable pharyngeal positioning for imaging, which limits the generalizability of the model to younger pediatric populations. Finally, as the study coincided with the predominance of the Omicron JN.1 lineage in Japan, the model’s performance should be monitored for potential changes in pharyngeal manifestations with future variants.

### Conclusions

In conclusion, the 3-tier COVID-19-AI facilitates clinical triage by stratifying patients into 3 actionable risk categories: high, medium, or low suspicion—formally designated as COVID-19 detected, suspended, or undetected, respectively. Our findings demonstrate that this noninvasive tool can achieve high sensitivity for ruling out infection and high specificity for ruling it in, potentially reducing the testing burden and procedural risks. We anticipate that widespread clinical adoption of this triage model will streamline infection control workflows and support clinicians in optimizing diagnostic resource allocation.

## Supplementary material

10.2196/87705 Multimedia Appendix 1 Supplementary methods for SARS-CoV-2 testing workflow and AI model development.

10.2196/87705 Multimedia Appendix 2 Participating institutions.

10.2196/87705 Multimedia Appendix 3 Predicted predictive values across different prevalence scenarios.

## Abbreviations

AI: artificial intelligence
AUROC: area under the receiver operating characteristic curve
RT-PCR: reverse transcription polymerase chain reaction
