# Supplementary material for: A 3-Tier AI Model for COVID-19 Triage Using Pharyngeal Images: Algorithm Development and Validation
Source: JMIR Form Res. 2026 Jul 20;10:e87705. doi: 10.2196/87705 (PMC13384471; doi:10.2196/87705)
Supplement: Multimedia Appendix 1 [file formative-v10-e87705-s001.docx]

***Multimedia Appendix 1. Supplementary methods.***

**SARS-CoV-2 Testing Workflow**

SARS-CoV-2 infection status was determined through site-based rapid antigen test and centralized RT-PCR analysis. Rapid antigen test was performed at each of the 26 participating institutions at the time of the initial visit. Ten types of kits, all approved as in vitro diagnostics by the Ministry of Health, Labour and Welfare of Japan, were used according to the real-world clinical setting at each site: (1) Quick Navi™-Flu+COVID19 Ag, (2) SARS-CoV-2 & Flu A/B Rapid Antigen Test, (3) Immuno Ace® SARS-CoV-2/Flu, (4) Immuno Ace® SARS-CoV-2 II, (5) Quick Chaser® SARS-CoV-2/Flu A,B, (6) V Trust SARS-CoV-2+Flu Ag, (7) Rapidtesta FLU & SARS-CoV-2, (8) Panbio™ COVID-19 A&B Rapid Panel, (9) Adtest SARS-CoV-2/Flu, and (10) Clinitest® Rapid COVID-19 Antigen. All specimens were nasopharyngeal swabs collected by trained physicians or nurses. The mean time from the first symptom onset to the site visit was 35.9 ± 30.7 hours, and specimen sampling was performed on the same day as the pharyngeal image acquisition during the patient's visit.

For the gold-standard reference, centralized RT-PCR was performed. Residual liquid from the rapid antigen test kits was frozen and transported to a central laboratory (Mediford Corporation, Tokyo, Japan). RNA was extracted using the QIAamp Viral RNA Mini Kit (Qiagen), and RT-PCR was conducted on the 7500 Fast Dx Real-Time PCR instrument (Life Technologies Japan) using the TRexGene® SARS-CoV-2 & Flu A/B Detection Kit (Toyobo Co., Ltd.). The amplification protocol consisted of 45 cycles. A sample was considered positive if a *Ct* value was detected within 45 cycles. Internal controls were required to have a *Ct* ≤ 40 for a valid run.

**Development of the AI Model to Predict COVID-19 Diagnosis**

We developed an ensemble AI model (version COV2025.10) to predict the probability of COVID-19, using pharyngeal images and clinical information (COVID-19-AI). For the model development (training) phase, "COVID-19 positive" was defined as being confirmed by both RT-PCR and rapid antigen test, while all other cases (including discordant results) were treated as negative. This model consists of two machine learning components: a Shifted Window Transformer (Swin Transformer) and a boosting model. During the training phase, we sequentially connected these two models, using the output of the first image-based model as the input to the second model, thereby constructing a stacked ensemble AI model. Python (version 3.12.8; Python Software Foundation) was used for Al model development and statistical analysis.

**Data Partitioning and Cross-Validation**

The multicenter, prospectively collected dataset was divided into a training set (n=2,133) and a separate test set (n=696). To enhance model robustness across diverse clinical presentations, the data were partitioned based on a combination of participating institutions and eight distinct diagnostic patterns (reflecting all possible combinations of positive/negative results for COVID-19, Influenza A, and Influenza B). For the training set, we employed stratified 5-fold cross-validation to optimize the model while ensuring that the prevalence of COVID-19 remained consistent across all folds. Among data from 2,133 patients used to train the COVID-19-AI model, 1,064 (49.9%) were male and the mean age was 31.3 years (SD 18.8); 773 (36.4%) were RT-PCR-positive for COVID-19 (**Supplementary Table 1**). Using this training set, we developed an ensemble AI model to estimate the probability of SARS-CoV-2 infection at the individual level.

**Image Processing and Model Training**

For each patient, a single representative pharyngeal image was used for analysis. To quantify the visual image quality criteria, we utilized an automated evaluation system based on a lightweight CNN model [1]. This system was trained using human-annotated visual image quality criteria (e.g., visibility of the posterior pharyngeal wall, brightness, focus, motion blur, and exhalation fog) defined by one of the authors (MF), who is a physician. Using this system, the best-quality image for each patient was selected from the captured frames and used as the model input. This automated selection process ensures that high-quality data is consistently used for all participants, thereby preventing selection bias associated with the manual exclusion of technically inadequate images.

The input images for the Swin-Transformer were resized and subjected to data augmentation (e.g., flipped, rotated, blurred, and contrast-changed) to improve the model's accuracy and generalization performance. To prevent overfitting, we used well-established training strategies, including batch normalization, learning rate decay, and stratified cross-validation. These methods ensure that the model remains robust across diverse clinical settings.

**Feature Importance and Performance Evaluation**

To evaluate the contribution of each input feature to the model's predictions, we used the built-in feature importance methods of CatBoost and LightGBM, which calculate the average change in the prediction value when a feature is used for splitting in decision trees. The feature-importance analysis of each variable in the model indicated that pharyngeal images were the dominant contributors to model predictions, surpassing basic clinical information (**Supplementary Figure 1**). To evaluate the specific contribution of pharyngeal imaging, we also conducted an ablation study comparing the performance of the integrated model (clinical information and pharyngeal images) against models using only clinical information or only pharyngeal images. The area under the receiver operating characteristic curve (AUROC) for the integrated model was 0.78 (95%CI, 0.76-0.80), which significantly outperformed that of the model using only clinical information (AUROC, 0.75; 95%CI, 0.73-0.77; *P* < .001) or the model using only pharyngeal images (AUROC, 0.71; 95%CI, 0.69-0.73; *P* < .001) (**Supplementary Figure 2**). These findings confirm that the inclusion of pharyngeal image information provides significant incremental predictive value over traditional clinical data.

**Three-tier Output**

The COVID-19-AI produces a three-tier output (High/Medium/Low) based on pharyngeal images and clinical variables. The thresholds for this classification were pre-defined during internal validation based on consultations with the Pharmaceuticals and Medical Devices Agency (PMDA) and clinician needs. Specifically, the boundary between ‘High/Medium’ and 'Low' was set to target a sensitivity of ≥80% for ruling out infection, while the boundary between 'High' and 'Medium/Low' was established to target a specificity of ≥90% for ruling in infection. These fixed thresholds were subsequently applied to the test set (i.e., performance evaluation cohort) for independent performance evaluation.

**Supplementary Table 1. Patient characteristics of the training data set (n = 2,133)**

| **Characteristics** | **Value** |
| --- | --- |
| Age (years), mean (SD) | 31.3 (18.8) |
| Sex, male / female, n (%) | 1,064 (49.9) / 1,069 (50.1) |
| Race, Asian / White / Black or African American / others, n (%) | 2,126 (99.7) / 2 (0.1) / 1 (0.0) / 4 (0.2) |
| Time from onset (hours), mean (SD)* | 35.8 (32.5) |
| Highest BT before visit (°C), mean (SD) | 38.3 (0.9) |
| Close contact with febrile patients within 14 days, n (%) | 532 (25.0) |
| Close contact with influenza patients within 3 days, n (%) | 234 (11.0) |
| Close contact with COVID-19 patients within 14 days, n (%) | 267 (12.5) |
| Recent influenza vaccination, n (%) | 757 (35.5) |
| Recent COVID-19 vaccination, n (%) | 1,481 (69.4) |
| Use of antipyretics, n (%) | 1,084 (50.8) |
| Subjective symptoms, n (%) |  |
| Joint pain | 601 (28.2) |
| Muscle pain | 302 (14.2) |
| Headache | 1,231 (57.7) |
| Tiredness | 1,373 (64.4) |
| Appetite loss | 379 (17.8) |
| Chill | 1,017 (47.7) |
| Sweating | 286 (13.4) |
| Cough | 1,266 (59.4) |
| Sore throat | 1,385 (64.9) |
| Nasal discharge | 1,139 (53.4) |
| Nasal congestion | 684 (32.1) |
| Abdominal pain | 139 (6.5) |
| Vomiting | 101 (4.7) |
| Diarrhea | 121 (5.7) |
| Sputum | 636 (29.8) |
| Dyspnea | 143 (6.7) |
| Smell disorder | 25 (1.2) |
| Taste disorder | 31 (1.5) |
| Objective findings |  |
| BT at visit (°C), mean (SD) | 37.5 (0.9) |
| Pulse rate (beats/min), mean (SD) | 98.2 (18.1) |
| Oxygen saturation (%), mean (SD) | 97.8 (1.2) |
| Tonsillitis, n (%) | 184 (8.6) |
| Tonsillar white moss, n (%) | 68 (3.2) |
| Tonsillar redness, n (%) | 753 (35.4) |
| Tonsillar swelling or exudate, n (%) | 43 (2.0) |
| Tender anterior cervical lymphadenopathy, n (%) | 40 (2.0) |
| Environment for pharyngeal imaging, n (%) |  |
| Indoor space without windows | 778 (36.5) |
| Indoor space with windows | 1,100 (51.6) |
| Covered outdoor area | 253 (11.9) |
| Uncovered outdoor area | 2 (0.1) |
| Influenza test (rapid antigen test), + / −, n (%) | 648 (30.4) / 1,485 (69.6) |
| Influenza test (RT-PCR), + / −, n (%) | 755 (35.5) / 1,371 (64.5) |
| COVID-19 test (rapid antigen test), + / − / indeterminate, n (%) | 617 (28.9) / 1,515 (71.0) / 1 (0.0) |
| COVID-19 test (RT-PCR), + / −, n (%) | 773 (36.4) / 1,353 (63.6) |

**Note.**

*Time from symptom onset to the study site visit.

Abbreviations: BT, body temperature; RT-PCR, reverse transcription-polymerase chain reaction.


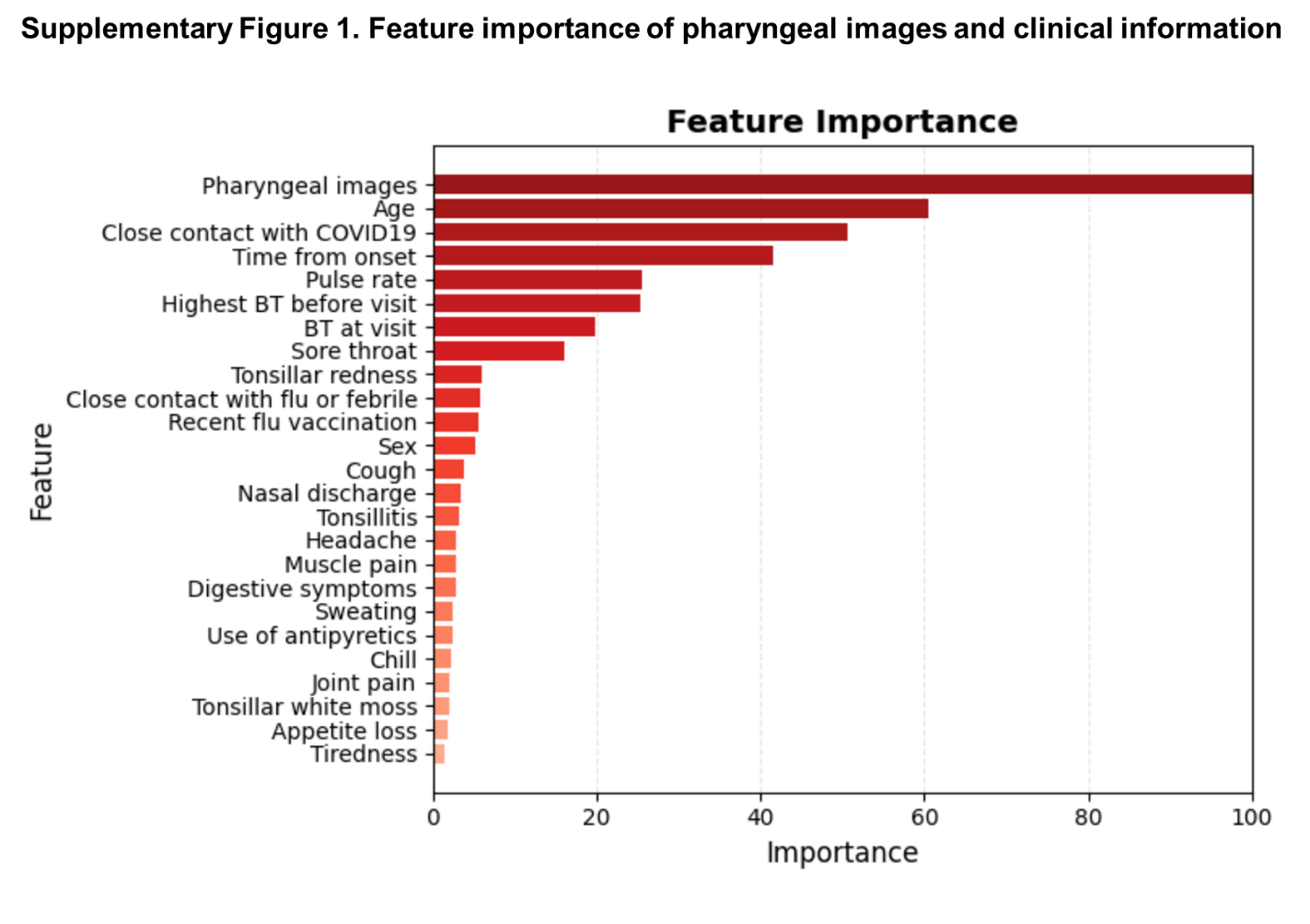


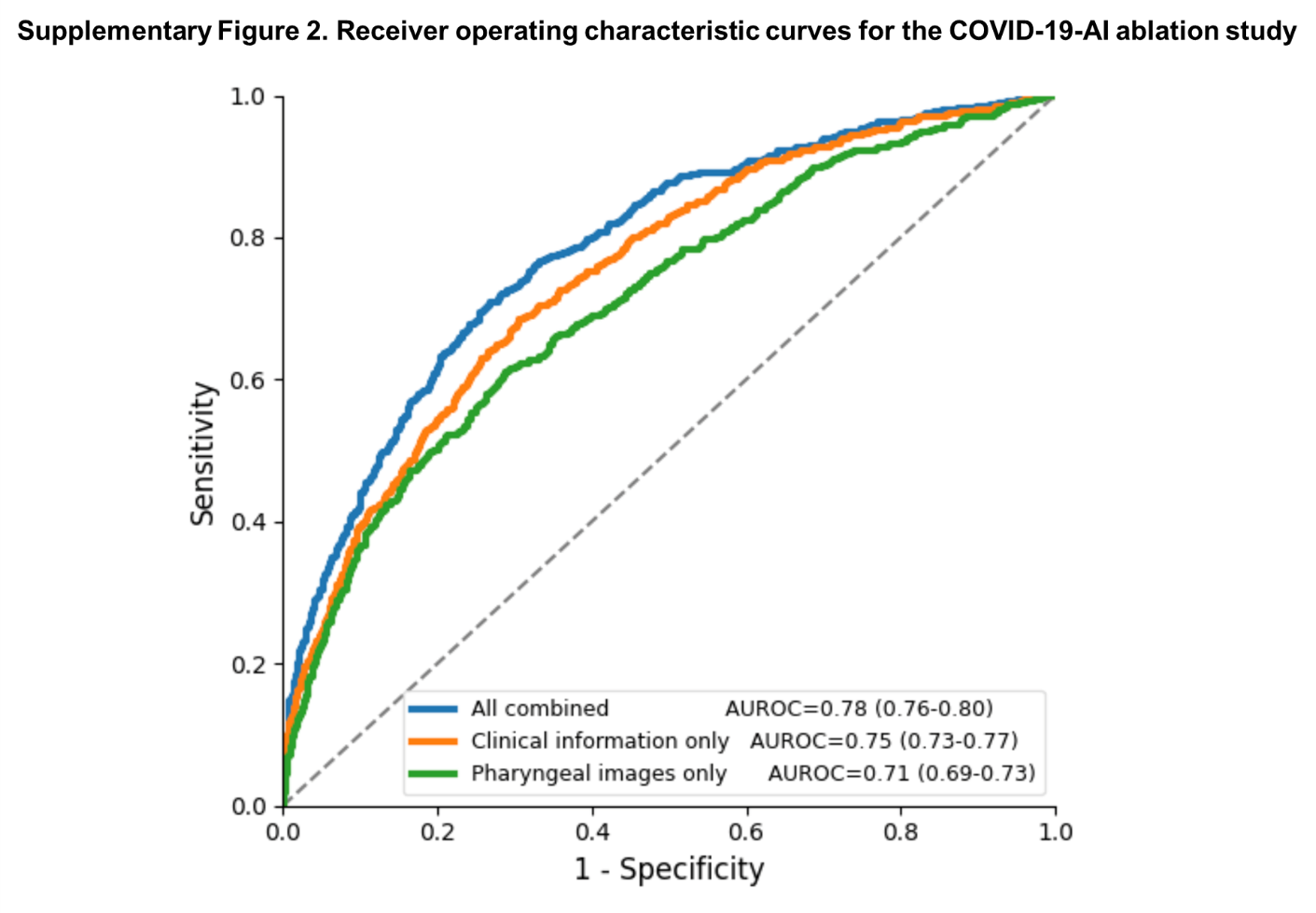


**References**

1. Sandler M, Howard A, Zhu M, Zhmoginov A, Chen LC. MobileNetV2: inverted residuals and linear bottlenecks. In: Proceedings of the IEEE/CVF Conference on Computer Vision and Pattern Recognition. 2018 Presented at: IEEE/CVF Conference on Computer Vision and Pattern Recognition; Jun 18-23, 2018; Salt Lake City, UT, USA URL: https://ieeexplore.ieee.org/document/8578572/ [doi: 10.1109/cvpr.2018.00474]
