# Supplementary material for: A 3-Tier AI Model for COVID-19 Triage Using Pharyngeal Images: Algorithm Development and Validation
Source: JMIR Form Res. 2026 Jul 20;10:e87705. doi: 10.2196/87705 (PMC13384471; doi:10.2196/87705)
Supplement: Multimedia Appendix 2 [file formative-v10-e87705-s002.docx]

***Multimedia Appendix 2. Participating institutions.***

**Supplementary Table 2. Characteristics of the 26 Participating Institutions**

| No. | Institution Name | Type | Specialty | Region |
| --- | --- | --- | --- | --- |
| 1 | Fukuda Internal Medicine Clinic | Clinic | Internal Medicine / Pediatrics | Kanto |
| 2 | Kanjamesen Clinic Ginza-Shinbashi | Clinic | Internal Medicine | Kanto |
| 3 | Kanjamesen Clinic Toranomon | Clinic | Internal Medicine | Kanto |
| 4 | Yoshida Clinic | Clinic | Internal Medicine | Kanto |
| 5 | Keiyu Ginza Clinic | Clinic | Otolaryngology (ENT) | Kanto |
| 6 | Yamaguchi Clinic | Clinic | Internal Medicine | Kanto |
| 7 | Shu Clinic | Clinic | Internal Medicine | Kanto |
| 8 | Onaka Internal Medicine Higashi-shirakabe Clinic | Clinic | Internal Medicine | Central |
| 9 | Takahashi Clinic | Clinic | Pediatrics | Central |
| 10 | Kirakira Kodomo Clinic | Clinic | Pediatrics | Central |
| 11 | Moriya Pediatrics Clinic | Clinic | Pediatrics | Shikoku |
| 12 | Irie Internal Medicine and Pediatrics Clinic | Clinic | Internal Medicine / Pediatrics | Shikoku |
| 13 | So Otolaryngology Clinic | Clinic | Otolaryngology (ENT) | Shikoku |
| 14 | Funai Otolaryngology Clinic | Clinic | Otolaryngology (ENT) | Kyushu |
| 15 | Aozora Pediatrics Clinic | Clinic | Pediatrics | Kyushu |
| 16 | Kamoike Otolaryngology Clinic | Clinic | Otolaryngology (ENT) | Kyushu |
| 17 | Moriyama Otolaryngology Clinic | Clinic | Otolaryngology (ENT) | Kyushu |
| 18 | Adachi Kyosai Hospital | Hospital | Internal Medicine / General Practice | Kanto |
| 19 | Kamei Internal Medicine and Respiratory Clinic | Clinic | Internal Medicine | Hokkaido |
| 20 | Tashiro Metabolic Internal Medicine Clinic | Clinic | Internal Medicine | Kyushu |
| 21 | Miyanosawa Internal Medicine and Cardiology Clinic | Clinic | Internal Medicine | Hokkaido |
| 22 | Otolaryngology Mick | Clinic | Otolaryngology (ENT) | Kyushu |
| 23 | Ugumori Otolaryngology Clinic | Clinic | Otolaryngology (ENT) | Kyushu |
| 24 | Murasakibaru Tahara Clinic | Clinic | Pediatrics | Kyushu |
| 25 | Bandai Medical Clinic | Clinic | Internal Medicine | Kyushu |
| 26 | Shuwa Otolaryngology Clinic | Clinic | Otolaryngology (ENT) | Kyushu |
