# Supplementary material for: A 3-Tier AI Model for COVID-19 Triage Using Pharyngeal Images: Algorithm Development and Validation
Source: JMIR Form Res. 2026 Jul 20;10:e87705. doi: 10.2196/87705 (PMC13384471; doi:10.2196/87705)
Supplement: Multimedia Appendix 3 [file formative-v10-e87705-s003.docx]

***Multimedia Appendix 3. Predicted Predictive Values Across Different Prevalence Scenarios.***

**Supplementary Table 3. Predicted predictive values (Rule-in and Rule-out capabilities) across various COVID-19 prevalence scenarios.**

| Prevalence | Criteria* | Sensitivity | Specificity | PPV  (Rule-in capability) | NPV  (Rule-out capability) |
| --- | --- | --- | --- | --- | --- |
| 5% (Screening) | Inclusive | 93.9% | 19.6% | 5.8% | 98.4% |
|  | Strict | 24.7% | 94.4% | 18.8% | 96.0% |
| 10% (Screening) | Inclusive | 93.9% | 19.6% | 11.5% | 96.7% |
|  | Strict | 24.7% | 94.4% | 32.9% | 91.9% |
| 35.5% (Actual Study) | Inclusive | 93.9% | 19.6% | 39.1% | 85.4% |
|  | Strict | 24.7% | 94.4% | 70.9% | 69.5% |

**Note.**

Actual study prevalence was 35.5% (247/696).

Calculations for the 5% and 10% scenarios are based on the diagnostic performance observed in the full analysis set (n = 696).

* Inclusive criteria: High or Medium suspicion (Positive). Strict criteria: High suspicion (Positive).

Abbreviations: PPV, Positive Predictive Value; NPV, Negative Predictive Value.
